# Supplementary material for: Human Gait Activity Recognition Machine Learning Methods
Source: Sensors (Basel). 2023 Jan 9;23(2):745. doi: 10.3390/s23020745 (PMC9865094; doi:10.3390/s23020745)
Supplement: Supplementary file 1 [file sensors-23-00745-s001.zip › ML_full_parameters.pdf]

Table S1. ML algorithm full parameters.

Classifier algorithm	Algorithm's hyper-parameters for CWT dataset	Algorithm's hyper-parameters for RAW dataset
ANN	Number of learnable parameters= 8.4k,<br>Solver= 'ADAM',<br>Mini batch size= 64,<br>Shuffle= 'every-epoch',<br>Max epoch= 50,<br>Learn Rate= 0.01,<br>LR drop period= 10,<br>LR drop factor= 0.9,<br>Output network= 'best validation loss'.<br>*Layers:*<br>*Sequence input (540);*<br>*Fully connected (16);*<br>*Batch normalization;*<br>*Fully connected (5);*<br>*Batch normalization;*<br>*Softmax;*<br>*Classification;*	Number of learnable parameters= 463,<br>Solver= 'ADAM',<br>Mini batch size= 64,<br>Shuffle= 'every-epoch',<br>Max epoch= 50,<br>Learn Rate= 0.01,<br>LR drop period= 10,<br>LR drop factor= 0.9,<br>Output network= 'best validation loss'.<br>*Layers:*<br>*Sequence input (20);*<br>*Fully connected (16);*<br>*Batch normalization;*<br>*Fully connected (5);*<br>*Batch normalization;*<br>*Softmax;*<br>*Classification;*
DT	Max. epochs= 50,<br>Number of learnable parameters= 2,<br>Max. Num. splits= 10,<br>Min. Parent size= 2,<br>Num. grid divisions= 10,<br>KFold= 10,<br>Surrogate= 'off'<br>Bayesian optimization of split criterion and min. leaf size.	Max. epochs= 50,<br>Number of learnable parameters= 2,<br>Max. Num. splits= 10,<br>Min. Leaf size= 1,<br>Min. Parent size= 2,<br>Num. grid divisions= 10,<br>KFold= 10,<br>Surrogate= 'off'<br>Bayesian optimization of split criterion.
SVM	Coding= 'onevsone',<br>Learners= 'SVM',<br>Number of learnable parameters=6,<br>Max epoch= 7,<br>KFold= 4,<br>Class prior probabilities= 'empirical'<br>Bayesian optimization of Box constraint and kernel size.	Coding= 'onevsone',<br>Learners= 'SVM',<br>Number of learnable parameters=6,<br>Max epoch= 7,<br>KFold= 4,<br>Class prior probabilities= 'empirical'<br>Bayesian optimization of Box constraint and kernel size.
NB model	Data distribution= 'gaussian',<br>Kernel smoother type= 'normal',<br>Support= 'unbounded',<br>KFold= 10,<br>Prior probabilities= 'empirical'.	Data distribution= 'gaussian',<br>Kernel smoother type= 'normal',<br>Support= 'unbounded',<br>KFold= 10,<br>Prior probabilities= 'empirical'.
LSTM NN	Number of learnable parameters= 410k,<br>LSTM Solver= 'SGDM',<br>Mini batch size= 16,<br>Shuffle= 'every-epoch',<br>Max epoch= 60,<br>Learn Rate= 0.05,<br>Output network= 'best validation loss'.	Number of learnable parameters= 6.9k<br>LSTM Solver= 'SGDM',<br>Mini batch size= 16,<br>Shuffle= 'every-epoch',<br>Max epoch= 60,<br>Learn Rate= 0.05,<br>Output network= 'best validation loss'.
LSTM NN	*Layers:*<br>*Sequence input (540);*<br>*biLSTM (64);*<br>*Relu;*<br>*Batch normalization;*<br>*Dropout (0.5);*<br>*biLSTM (64);*<br>*Relu;*<br>*Batch normalization;*<br>*Dropout (0.5);*<br>*Fully connected (5);*<br>*Batch normalization;*<br>*Softmax;*<br>*Classification;*	*Layers:*<br>*Sequence input(20);*<br>*biLSTM (12);*<br>*Relu;*<br>*Batch normalization;*<br>*Dropout (0.5);*<br>*biLSTM (12);*<br>*Relu;*<br>*Batch normalization;*<br>*Dropout (0.5);*<br>*Fully connected (5);*<br>*Batch normalization;*<br>*Softmax;*<br>*Classification;*
AE+Softmax	Number of bottleneck neurons= 100,<br>Number of softmax neurons= 5,	Number of bottleneck neurons= 12,<br>Number of softmax neurons= 5,

AE+Softmax	Number of learnable parameters= 109k,<br>Solver= 'Scaled conjugate gradient',<br>Encoder Max epoch= 400,<br>Softmax Max epoch= 400,<br>Encoder transfer function= logsig,,<br>Decoder transfer function= logsig,<br>L2 weight regularization= 0.001,<br>Loss function= MSE sparse,<br>Sparsity proportion= 0.05,<br>Sparsity regularization= 1.	Number of learnable parameters= 522,<br>Solver= 'Scaled conjugate gradient',<br>Encoder Max epoch= 400,<br>Softmax Max epoch= 400,<br>Encoder transfer function= logsig,,<br>Decoder transfer function= logsig,<br>L2 weight regularization= 0.001,<br>Loss function= MSE sparse,<br>Sparsity proportion= 0.05,<br>Sparsity regularization= 1.
AE+biLSTM NN	Encoder bottleneck neurons= 100,<br>Number of learnable parameters= 171k,<br>Encoder Max epoch= 400,<br>Encoder transfer function= logsig,<br>Decoder transfer function= logsig,<br>L2 weight regularization= 0.001,<br>Loss function= MSE sparse,<br>Sparsity proportion= 0.05,<br>Sparsity regularization= 1,<br>LSTM Solver= 'ADAM',<br>Mini batch size= 8,<br>Shuffle= 'every-epoch',<br>Max epoch= 75,<br>Learn Rate= 0.05,<br>Output network= 'best validation loss'.<br>*Layers:*<br>*Sequence input (540);*<br>*biLSTM (32);*<br>*Relu;*<br>*Batch normalization;*<br>*Dropout (0.5);*<br>*biLSTM (32);*<br>*Relu;*<br>*Batch normalization;*<br>*Dropout (0.5);*<br>*Fully connected (5);*<br>*Batch normalization;*<br>*Softmax;*<br>*Classification;*	Encoder bottleneck neurons= 12,<br>Number of learnable parameters= 60k,<br>Encoder Max epoch= 400,<br>Encoder transfer function= logsig,<br>Decoder transfer function= logsig,<br>L2 weight regularization= 0.001,<br>Loss function= MSE sparse,<br>Sparsity proportion= 0.05,<br>Sparsity regularization= 1,<br>LSTM Solver= 'ADAM',<br>Mini batch size= 8,<br>Shuffle= 'every-epoch',<br>Max epoch= 75,<br>Learn Rate= 0.05,<br>Output network= 'best validation loss'.<br>*Layers:*<br>*Sequence input (20);*<br>*biLSTM (32);*<br>*Relu;*<br>*Batch normalization;*<br>*Dropout (0.5);*<br>*biLSTM (32);*<br>*Relu;*<br>*Batch normalization;*<br>*Dropout (0.5);*<br>*Fully connected (5);*<br>*Batch normalization;*<br>*Softmax;*<br>*Classification;*
CNN+RNN	Num. of learnable parameters= 9.4M,<br>Solver= 'RMSPROP,<br>Mini batch size= 64,<br>Shuffle= 'every-epoch',<br>Max epoch= 50,<br>Learn Rate= 0.01,<br>LR drop period= 10,<br>LR drop factor= 0.9,<br>Output network= 'best validation loss'.<br>*Layers:*<br>*Sequence input(540);*<br>*1D convolution (filt.Size=64; num.filt.=256);*<br>*1D MaxPool (8);*<br>*Batch normalization;*<br>*Relu;*<br>*1D convolution (filt.size=32; num.filt.=64);*<br>*1D MaxPool (8);*<br>*Batch normalization;*<br>*Relu;*<br>*1D convolution (filt.Size=16; num.filt.=32);*<br>*1D MaxPool (8);*<br>*Batch normalization;*<br>*Relu;*<br>*Fully connected (8);*<br>*Batch normalization;*<br>*Fully connected (5);*<br>*Batch normalization;*<br>*Softmax;*<br>*Classification;*	Num. of learnable parameters= 15k,<br>Solver= 'RMSPROP,<br>Mini batch size= 64,<br>Shuffle= 'every-epoch',<br>Max epoch= 50,<br>Learn Rate= 0.01,<br>LR drop period= 10,<br>LR drop factor= 0.9,<br>Output network= 'best validation loss'.<br>*Layers:*<br>*Sequence input(20);*<br>*1D convolution (filt.Size=8; num.filt.=16);*<br>*1D MaxPool (6);*<br>*Batch normalization;*<br>*Relu;*<br>*1D convolution (filt.size=16; num.filt.=16);*<br>*1D MaxPool (4);*<br>*Batch normalization;*<br>*Relu;*<br>*1D convolution (filt.Size=32; num.filt.=16);*<br>*1D MaxPool (4);*<br>*Batch normalization;*<br>*Relu;*<br>*Fully connected (8);*<br>*Batch normalization;*<br>*Fully connected (5);*<br>*Batch normalization;*<br>*Softmax;*<br>*Classification;*

CNNA+RNN	Num. of learnable parameters= 47M,<br>Parallel CNN streams= 5,<br>Solver= 'RMSPROP,<br>Mini batch size= 64,<br>Shuffle= 'every-epoch',<br>Max epoch= 50,<br>Learn Rate= 0.01,<br>LR drop period= 10,<br>LR drop factor= 0.9,<br>Output network= 'best validation loss'.<br><br>*Layers:*<br>*Sequence input(540);*<br>*1D convolution (filt.Size=64; num.filt.=256);*<br>*1D MaxPool (8);*<br>*Batch normalization;*<br>*Relu;*<br>*1D convolution (filt.size=32; num.filt.=64);*<br>*1D MaxPool (8);*<br>*Batch normalization;*<br>*Relu;*<br>*1D convolution (filt.Size=16; num.filt.=32);*<br>*1D MaxPool (8);*<br>*Batch normalization;*<br>*Relu;*<br>*ABOVE LAYERS ARE USED FIVE TIMES, WIRED IN PARALLEL*<br>*Add (5);*<br>*Fully connected (8);*<br>*Batch normalization;*<br>*Fully connected (5);*<br>*Batch normalization;*<br>*Softmax;*<br>*Classification;*	Num. of learnable parameters= 75k,<br>Parallel CNN streams= 5,<br>Solver= 'RMSPROP,<br>Mini batch size= 64,<br>Shuffle= 'every-epoch',<br>Max epoch= 50,<br>Learn Rate= 0.01,<br>LR drop period= 10,<br>LR drop factor= 0.9,<br>Output network= 'best validation loss'.<br><br>*Layers:*<br>*Sequence input(20);*<br>*1D convolution (filt.Size=8; num.filt.=16);*<br>*1D MaxPool (6);*<br>*Batch normalization;*<br>*Relu;*<br>*1D convolution (filt.size=16; num.filt.=16);*<br>*1D MaxPool (4);*<br>*Batch normalization;*<br>*Relu;*<br>*1D convolution (filt.Size=32; num.filt.=16);*<br>*1D MaxPool (4);*<br>*Batch normalization;*<br>*Relu;*<br>*ABOVE LAYERS ARE USED FIVE TIMES, WIRED IN PARALLEL*<br>*Fully connected (8);*<br>*Batch normalization;*<br>*Fully connected (5);*<br>*Batch normalization;*<br>*Softmax;*<br>*Classification;*
