# Supplementary material for: Human Gait Activity Recognition Machine Learning Methods
Source: Sensors (Basel). 2023 Jan 9;23(2):745. doi: 10.3390/s23020745 (PMC9865094; doi:10.3390/s23020745)

Journal name: MDPI Sensors 2022

Authors and title: Jan Slemenšek, Iztok Fister, Jelka Geršak, Božidar Bratina, Vesna Marija van Midden, Zvezdan Pirtošek, Riko Šafarič.

Human gait recognition machine learning methods

Total Number of files: 18.

Total size of files: 31.3 Mb.

Supplied supplementary material is divided into four categories:

- Raw dataset – Healthy subject.
- Matlab imported dataset – Healthy subject.
- Raw dataset – PD patient.
- Matlab imported dataset – PD patient.

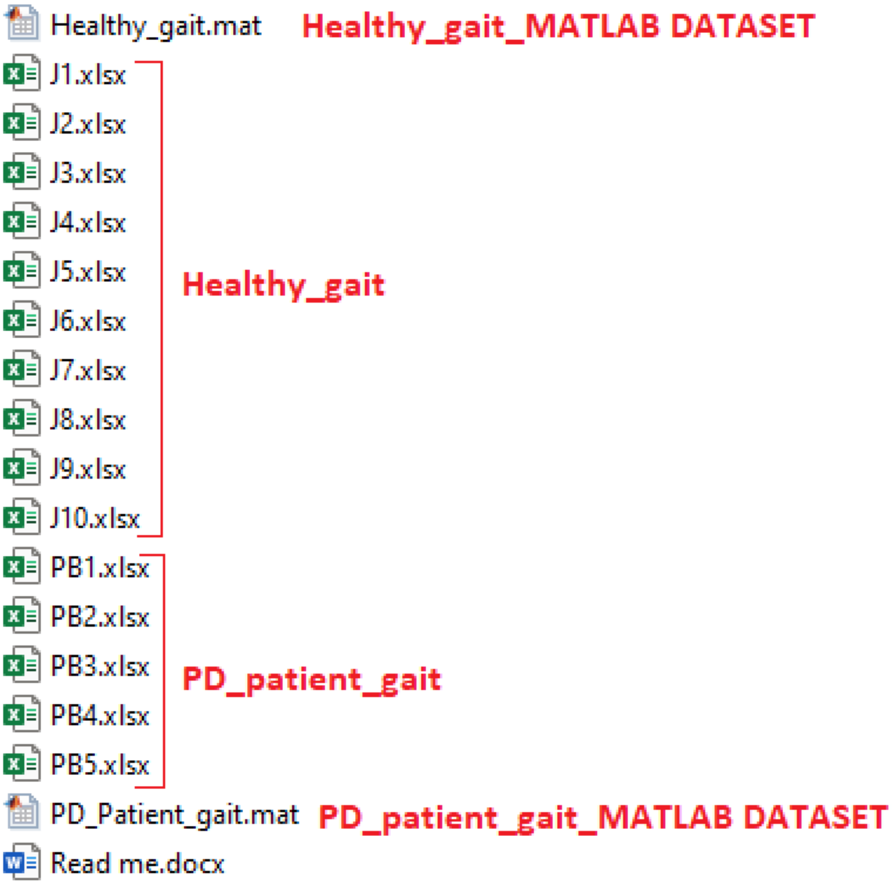

Supplement: Supplementary file 1 [file sensors-23-00745-s001.zip › Read me.pdf]
