# Supplementary figures and images for: Human Gait Activity Recognition Machine Learning Methods
Source: Sensors (Basel). 2023 Jan 9;23(2):745. doi: 10.3390/s23020745 (PMC9865094; doi:10.3390/s23020745)

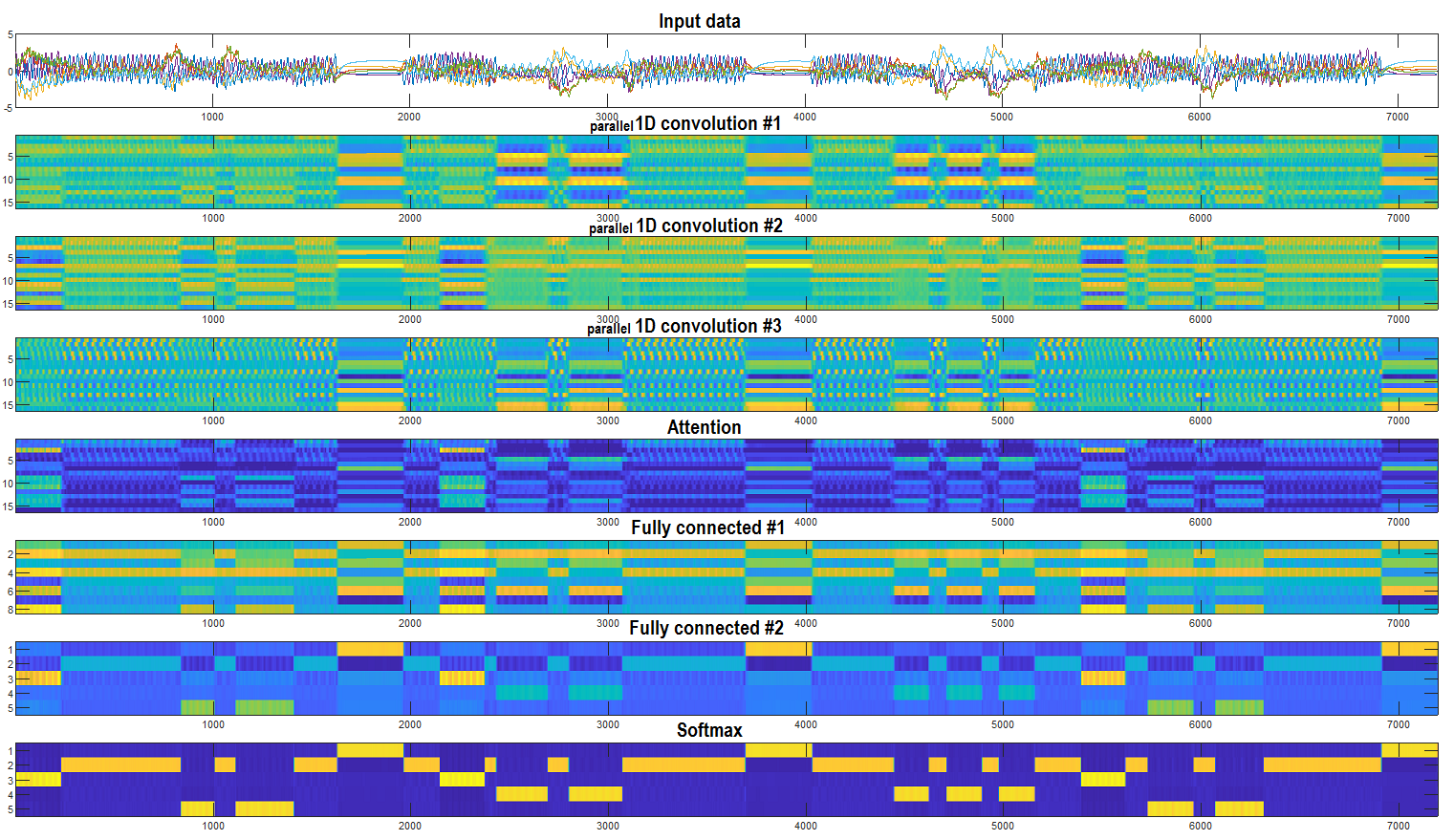

Supplement: Supplementary file 1 [file sensors-23-00745-s001.zip › CNNA+RNN_activations.png]
